# Blood pressure‐independent renoprotective effects of small interference RNA targeting liver angiotensinogen in experimental diabetes

**DOI:** 10.1111/bph.15955

**Published:** 2022-10-02

**Authors:** Edwyn O. Cruz‐López, Liwei Ren, Estrellita Uijl, Marian C. Clahsen‐van Groningen, Richard van Veghel, Ingrid M. Garrelds, Oliver Domenig, Marko Poglitsch, Ivan Zlatev, Timothy Rooney, Anne Kasper, Paul Nioi, Don Foster, A. H. Jan Danser

**Affiliations:** ^1^ Division of Vascular Medicine and Pharmacology, Department of Internal Medicine Erasmus MC Rotterdam The Netherlands; ^2^ Department of Pharmacy Shenzhen People's Hospital (The Second Clinical Medical College, Jinan University, The First Affiliated Hospital, Southern University of Science and Technology) Shenzhen China; ^3^ Division of Nephrology and Transplantation, Department of Internal Medicine Erasmus MC Rotterdam The Netherlands; ^4^ Department of Pathology Erasmus MC Rotterdam The Netherlands; ^5^ Institute of Experimental Medicine and Systems Biology University Hospital Aachen, RWTH Aachen University Aachen Germany; ^6^ Attoquant Diagnostics Vienna Austria; ^7^ Alnylam Pharmaceuticals Cambridge Massachusetts USA

**Keywords:** diabetes, hypertension, renin‐angiotensin system, small interfering RNA

## Abstract

**Background and Purpose:**

Small interfering RNA (siRNA) targeting liver angiotensinogen lowers blood pressure, but its effects in hypertensive diabetes are unknown.

**Experimental Approach:**

To address this, TGR (mRen2)27 rats (angiotensin II‐dependent hypertension model) were made diabetic with streptozotocin over 18 weeks and treated with either vehicle, angiotensinogen siRNA, the AT_1_ antagonist valsartan, the ACE inhibitor captopril, valsartan + siRNA or valsartan + captopril for the final 3 weeks. Mean arterial pressure (MAP) was measured via radiotelemetry.

**Key Results:**

MAP before treatment was 153 ± 2 mmHg. Diabetes resulted in albuminuria, accompanied by glomerulosclerosis and podocyte effacement, without a change in glomerular filtration rate. All treatments lowered MAP and cardiac hypertrophy, and the largest drop in MAP was observed with siRNA + valsartan. Treatment with siRNA lowered circulating angiotensinogen by >99%, and the lowest circulating angiotensin II and aldosterone levels occurred in the dual treatment groups. Angiotensinogen siRNA did not affect renal angiotensinogen mRNA expression, confirming its liver‐specificity. Furthermore, only siRNA with or without valsartan lowered renal angiotensin I. All treatments lowered renal angiotensin II and the reduction was largest (>95%) in the siRNA + valsartan group. All treatments identically lowered albuminuria, whereas only siRNA with or without valsartan restored podocyte foot processes and reduced glomerulosclerosis.

**Conclusion and Implications:**

Angiotensinogen siRNA exerts renoprotection in diabetic TGR (mRen2)27 rats and this relies, at least in part, on the suppression of renal angiotensin II formation from liver‐derived angiotensinogen. Clinical trials should now address whether this is also beneficial in human diabetic kidney disease.

## Graphical abstract

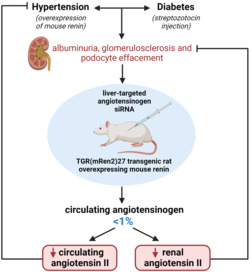

**Abbreviations**
MAP: mean arterial pressure
RAS: renin‐angiotensin‐system
TGR (mRen2)27: Transgenic rat (mRen2)27

**What is already known**

- Small interfering RNA (siRNA) targeting liver angiotensinogen lowers blood pressure in various hypertension models.

**What does this study add**

- Angiotensinogen siRNA exerts renoprotection in experimental diabetes by suppressing renal angiotensin formation from hepatic angiotensinogen.

**What is the clinical significance**

- Clinical trials should now investigate whether this approach is beneficial in human diabetic kidney disease.

## 1 INTRODUCTION

Hypertension is highly prevalent among diabetic patients (Deshpande et al., 2008; Epstein & Sowers, 1992). The association of these co‐morbidities is not surprising, since they share many common risk factors (Emdin et al., 2015; Tsimihodimos et al., 2018). Hypertension and diabetes also contribute synergistically to chronic kidney disease (Yamazaki et al., 2018). Diabetic kidney disease is a microvascular complication characterized by albuminuria and a reduced glomerular filtration rate (GFR). The percentage at which this occurs in diabetic patients increases when hypertension is present (Pantalone et al., 2015; Tuttle et al., 2014) and antihypertensive treatment helps to prevent renal damage (Raikou et al., 1998).

Conventional treatment for diabetic kidney disease often involves blockers of the renin‐angiotensin system (RAS), such as angiotensin‐converting enzyme inhibitors (ACEi) or AT_1_ receptor antagonists (also known as angiotensin II blockers [ARB]). Although these drugs decrease both blood pressure and albuminuria (Cryer et al., 2016), they do not always prevent the development of end‐stage renal disease.

A well‐known model to study the consequences of hypertension and diabetes is the diabetic transgenic rat (TGR) (mRen2)27 (Ren2 rat). These rats, which overexpress the mouse *Ren2* gene, are severely hypertensive (Campbell et al., 1995; Feldman et al., 2008). After streptozotocin injection, they develop a diabetic phenotype that closely mimics that observed in human diabetic patients, characterized by high levels of renin's precursor (prorenin), retinal pathology, vascular dysfunction and nephropathy (Batenburg et al., 2013; Conway et al., 2012; Hartner et al., 2007; Roksnoer et al., 2016; te Riet et al., 2014; Uijl et al., 2020; Yamaleyeva et al., 2012). Although prorenin's role is still unknown, one possibility is that it contributes to local angiotensin production in the kidney and other organs (Batenburg & Danser, 2012). ACE inhibitors and AT_1_ antagonists lower blood pressure and improve albuminuria in this model, yet without preventing glomerulosclerosis (Mifsud et al., 2002; Roksnoer et al., 2016; Uijl et al., 2020). The renin inhibitor aliskiren does diminish glomerulosclerosis in this model, suggesting that the degree of RAS blockade (particularly at the level of the kidney) may be a crucial factor (Riet et al., 2014). Alternatively, RAS blockade might be combined with other renoprotective drugs like the neprilysin (neutral endopeptidase) inhibitor sacubitril to achieve histological improvement on top of blood pressure lowering via RAS‐independent pathways (Uijl et al., 2020).

A novel tool to potently suppress the renal RAS is *N*‐acetylgalactosamine (GalNAc)‐conjugated small interfering RNA (siRNA) targeting liver angiotensinogen (Mullick et al., 2017; Uijl et al., 2019). Earlier studies making use of this liver‐specific angiotensinogen siRNA have revealed that renal angiotensin production in a wide range of models (spontaneously hypertensive rat, 5/6th nephrectomy rat and the deoxycorticosterone acetate (DOCA)‐salt rat) fully depends on angiotensinogen of hepatic origin (Bovée et al., 2021; Uijl et al., 2019, 2021). Interestingly, angiotensinogen siRNA more strongly suppressed the renal RAS than the circulating RAS. As a consequence, it exerted renoprotection in a blood pressure‐independent manner in the 5/6th nephrectomy chronic kidney disease model (Bovée et al., 2021).

In the present study we set out to evaluate this novel treatment tool in the diabetic Ren2 rat, making a comparison versus both an ACE inhibitor (captopril) and an AT_1_ antagonist (valsartan). To exclude the possibility of insufficient RAS blockade, we also tested two types of dual RAS blockade, that is, valsartan + captopril and valsartan + angiotensinogen siRNA. We hypothesized that angiotensinogen siRNA, either alone or in combination with valsartan, would lower blood pressure and improve albuminuria to at least the same degree as the classical RAS blockers (or their combination), while simultaneously improving renal microstructural damage, in particular podocyte integrity.

## 2 METHODS

### 2.1 Animal studies

All animal experiments were performed under the regulation and approval of the Animal Welfare Committee of the Erasmus MC (protocol number 17‐870‐01). Animal studies are reported in compliance with the ARRIVE guidelines (Percie du Sert et al., 2020) and with the recommendations made by the *British Journal of Pharmacology* (Lilley et al., 2020). Studies were designed to generate groups of equal size, using randomisation and blinded analysis. Given that the standard deviation of blood pressure in Ren2 rats equals 9.5 mmHg (unpublished observations), to aim at a minimum blood pressure difference of 20 mmHg (α = 0.05 and power = 0.8), a minimum of 7 animals/group is required. Male, heterozygous Ren2 rats (10‐week‐old; weight 300–500 g) were obtained by breeding homozygous Ren2 rats with Sprague–Dawley rats. Rats were maintained on a 12‐h light/dark cycle in type III cages (1 rat per cage) with access to standard rat chow food (Special Diets Services nr. 801727, Tecnilab‐bmi B.V., Someren, the Netherlands), and water *ad libitum*. Diabetes was induced by administering an intraperitoneal dose of streptozotocin (55 mg·kg^−1^), after which the animals were studied for 18 weeks. Following streptozotocin injection, non‐fasting blood glucose and β‐ketone levels were checked every 2 weeks (Precision Xceed, Abbott, Zwolle, the Netherlands). Only rats with glucose >15 mmol·L^−1^ were considered diabetic (Hartner et al., 2007) and they received 2 units of insulin (Levemir®). This was increased to a maximum of 5 units when β‐ketone levels were >2 mmol·L^−1^ and/or body weight decreased >6% in a week. During the final 3 weeks, rats were treated with either vehicle (n = 8), captopril (6 mg·kg^−1^·day^−1^; n = 8;), valsartan (4 mg·kg^−1^·day^−1^; n = 8), angiotensinogen siRNA (30 mg·kg^−1^ every 2 weeks via subcutaneous injection; n = 9), captopril + valsartan (n = 7), or angiotensinogen siRNA + valsartan (n = 7). The doses of the individual drugs were chosen because of their comparable blood pressure‐lowering effects in previous studies (Uijl et al., 2019, 2021; van Esch, Moltzer, et al., 2010). Valsartan and captopril were administered subcutaneously by osmotic mini‐pump (Alzet®, model 2ML4, Durect Corp, Cupertino, CA, USA). The siRNA consisted of a chemically modified antisense strand with sequence UUGAUUUUUGCCCAGGAUAGCUC, hybridized with a chemically modified sense strand of sequence GCUAUCCUGGGCAAAAAUCAA. Oligonucleotides were synthesized as previously described, with the *N*‐acetylgalactosamine ligand displayed at the 3′‐end of the siRNA sense strand (Uijl et al., 2019). Blood pressure, heart rate and activity were measured by radiotelemetry transmitters (HD‐S10, Data Sciences International, St. Paul, MN, USA) implanted 2 weeks before diabetes induction. Inhaled anaesthesia with isoflurane was applied at the time of radiotelemetry transmitter and mini‐pump implantation, as well as during intravenous injections and blood sample collection. Isoflurane was delivered using a precision vaporizer together with 20% oxygen (Sigma Delta, Penlon, UK), the isoflurane concentration amounting to 4%–5% in the induction chambers and 2%–3% in the face masks. Under anaesthesia, body temperature was maintained by using a heat therapy pump (T‐Pump, Stryker, MI, USA) connected to a heat pad on which the animals were placed during the entire procedure. For biochemical analysis, we collected 24‐h urine in metabolic cages and blood plasma by venipuncture from the lateral tail vein prior to the induction of diabetes mellitus (baseline) and at weeks 15 and 18 after injecting streptozotocin (pre‐treatment and post‐treatment, respectively). GFR measurements were performed at the same 3 time points. At the end of the 3 week‐treatment period, rats were anaesthetized by inhalation of isoflurane and exsanguinated. For angiotensin metabolites quantification 1 ml blood was collected in 10 ml of a 4 mol·L^−1^ guanidine thiocyanate solution (Campbell et al., 1999); remaining blood was collected in EDTA tubes and centrifuged to obtain plasma. Kidneys and heart were rapidly excised, weighed and divided into transverse segments that were either snap‐frozen in liquid nitrogen or preserved for histological analysis in 4% (w/v) paraformaldehyde or glutaraldehyde 1.2%‐paraformaldehyde 4%. Mesenteric and iliac arteries were isolated and used directly for myograph studies.

### 2.2 Biochemical measurements

The plasma concentrations of angiotensinogen, renin and total renin (the sum of renin and prorenin) were measured by enzyme‐kinetic assay as described before (de Lannoy et al., 1997; Uijl et al., 2019). Total plasma renin was measured after prorenin had been converted to renin by incubation of the sample for 48 h at 4°C with trypsin coupled to Sepharose (de Lannoy et al., 1997). Subtraction of the renin concentration from the total renin concentration yielded the prorenin concentration. The lower limits of detection of the angiotensinogen and renin assays were 0.2 pmol·ml^−1^ and 0.17 ng·ml^−1^·h^−1^, respectively. In the cases that measurements were at or below the lower limits of detection, this limit was applied to allow for statistical analysis. Plasma aldosterone was measured by solid‐phase radioimmunoassay (lower limit of detection 14.8 pg·ml^−1^; Diagnostic Products Corporation; Siemens Medical Solutions Diagnostics, Los Angeles, CA, U.S.A.). In hepatic and renal tissue, angiotensinogen was quantified by western blotting and normalized versus GAPDH as described before (Uijl et al., 2021), in accordance with BJP guidelines (Alexander et al., 2018). Angiotensin metabolites in plasma, kidney and heart tissue (left ventricle) were measured by liquid chromatography with tandem mass spectrometry analysis as described before (Roksnoer et al., 2015). Briefly, tissue samples were homogenized under liquid nitrogen and extracted with a guanidinium‐based extraction buffer. Stabilized whole blood and tissue extracts were spiked with stable isotope labelled internal standards for each individual target analyte (Sigma Aldrich) before being subjected to C18‐based solid phase extraction and subsequent liquid chromatography with tandem mass spectrometry analysis. The lower limit of quantification (LLOQ) in blood/tissue were 4/10 (angiotensin I), 2/8 (angiotensin II), 8/15 (angiotensin‐(1‐7)), 2/5 (angiotensin‐[1–5]), 2/5 (angiotensin‐[2–8]) and 2/10 (angiotensin‐[3–8]) pg·ml ^−1^ or g tissue, respectively. Urinary albumin was measured with a rat albumin ELISA kit (lower limit of detection 0.44 ng·ml^−1^; Abcam, Cambridge, UK). Plasma N‐terminal pro‐B‐type natriuretic peptide (NT‐proBNP) was measured with an ELISA (lower limit of detection 15.6 pg·ml^−1^; Aviva Systems Biology, San Diego, CA, USA). Plasma or serum potassium, sodium, glucose, insulin, creatinine, urea, alanine aminotransferase (ALT), aspartate aminotransferase (AST) and urinary sodium, potassium, creatinine and urea (24‐h urine) were measured at the clinical chemistry laboratory of the Erasmus MC.

### 2.3 Quantitative polymerase chain reaction

Total RNA was isolated from snap‐frozen kidney using the TRIzol Reagent (Sigma‐Aldrich) and reverse transcribed into cDNA using the QuantiTect Reverse Transcription Kit (Qiagen, Venlo, the Netherlands). cDNA amplification was done in duplicate in 40 cycles (denaturation at 95°C for 3 min, thermal cycling at 95°C for 3 s, annealing/extension at 60°C for 20 s) followed by a melt curve with a CFX384 (Bio‐Rad, Veenendaal, the Netherlands) using Kapa SYBR® Fast (Kapa Biosystems). Intron‐spanning oligonucleotide primers were designed with NCBI Primer‐BLAST (Table S1). The ΔΔCt method was used for relative quantification of mRNA expression levels. Angiotensinogen, renin, AT_1a_ receptor and AT_1b_ receptor were normalized versus the average of β_2_‐microglobulin and β‐actin; Neph1, nephrin and podocin were normalized versus the podocyte‐specific gene Wilms tumour‐1.

### 2.4 Kidney function

GFR was determined by transcutaneous measurement of fluorescein isothiocyanate‐labelled sinistrin clearance curves (Mannheim Pharma & Diagnostics GmbH, Mannheim, Germany) (Schock‐Kusch et al., 2009), administered as a bolus injection (0.24 mg·kg^−1^ dissolved in 0.9% saline solution) in the lateral tail vein. A noninvasive clearance–kidney fluorescent detection device and software (Mannheim Pharma & Diagnostics GmbH) was used to generate the elimination kinetics curve of fluorescein isothiocyanate‐sinistrin. GFR was derived from the excretion half‐life (t_1/2_) of fluorescein isothiocyanate‐sinistrin, using a conversion factor and formula validated for rats (Schock‐Kusch et al., 2009): GFR (ml·min^−1^ per 100 g body weight) = 31.26 (ml per 100 g body weight)/t_1/2_ fluorescein isothiocyanate‐sinistrin (min).

### 2.5 Histology

Kidney segments fixed in 4% paraformaldehyde were dehydrated and paraffin embedded. Transversal deparaffinized kidney sections (2 μm) were stained with periodic acid‐Schiff (PAS) and scored semi‐quantitatively in a blinded fashion by a renal pathologist (M. C. C. v. G.) as described previously (Uijl et al., 2020). Focal segmental glomerulosclerosis (FSGS) was assessed and graded in all glomeruli of one kidney section per rat, using an arbitrary scale in which 0%, <25%, 25%–50%, 50%–75% and >75% of glomerular sclerosis corresponded to grade 0 (n_0_), 1 (n_1_), 2 (n_2_), 3 (n_3_) and 4 (n_4_), respectively where (n) represents the number of glomeruli in each category. The glomerulosclerosis index (GSI) was calculated using the formula [(1 × n_1_) + (2 × n_2_) + (3 × n_3_) + (4 × n_4_)]/(n_0_ + n_1_ + n_2_ + n_3_ + n_4_). Tubular atrophy, interstitial fibrosis and tubulointerstitial inflammation were scored in the same kidney section and summed to obtain the tubulointerstitial score (TIS). A score of 0 to 3 indicates <25% of tubulointerstitial tissue damage, a score of 4 to 6 indicates 25%–50% and a score of 7 to 9 indicated >50%. Heart sections, fixed in 4% paraformaldehyde were dehydrated and paraffin‐embedded. Gomori silver staining was applied to deparaffinized left ventricle sections (5 μm) to visualize individual cardiomyocytes. Sirius red staining was used to visualize collagen as a measure of cardiac fibrosis. Cardiomyocyte area and collagen % were quantified using Qwin image analysis software (Leica, Cambridge, UK). Only transversally cut cells showing a nucleus were used to determine the cardiomyocyte area.

### 2.6 Electron microscopy

Kidney segments were fixed in a glutaraldehyde 1.2%–paraformaldehyde 4% solution. After fixation, kidney segments were embedded in epoxy resins in an automated tissue processor (Leica EM TP, Cambridge, UK) and cut in semi‐thin slices (1 μm) to check for tissue representation. After selection, ultra‐thin (60–70 nm) slices were cut and placed on 200 mesh uncoated transmission electron microscopy grids. Contrasting was in an automatic contrasting device for transmission electron microscopy grids (Leica AC20, Cambridge, UK). Pictures and interpretation of electron microscopy were obtained and scored in a blinded fashion by a renal pathologist (M. C. C. v. G.).

### 2.7 Statistics

The data and statistical analysis comply with the recommendations of the *British Journal of Pharmacology* on experimental design and analysis in pharmacology (Curtis et al., 2018). Data are expressed as mean ± SEM. Statistical analysis was undertaken only for studies where each group size was at least n = 5. The declared group size is the number of independent values and that statistical analysis was done using these independent values. Non‐normally distributed data were log‐transformed before statistical analysis. Data were analysed by one‐way ANOVA and mixed linear models, using treatment and time as fixed effects. If significant (F in ANOVA *P* < 0.05), selected post hoc analyses (Tukey's test, when among all groups; Dunnett's test, when comparing groups to control) were performed. Data obtained at multiple points in time were analysed using a repeated‐measures two‐way ANOVA, followed by post hoc correction according to Dunnett or Dunn in case of multiple comparisons, provided there was no significant variance inhomogeneity. Univariate linear associations were assessed by calculation of Pearson's coefficient of correlation. Two‐tailed *P* < 0.05 was considered statistically significant. Multiple linear regression analysis was performed to identify variables correlating independently. All analyses were performed using Prism, version 8.0.0 (GraphPad Software, Inc., La Jolla, California).

### 2.8 Materials

Streptozotocin was obtained from Merck Millipore, Amsterdam, the Netherlands. Insulin (Levemir®0 was obtained from Novo Nordisk, Copenhagen, Denmark. Captopril and guanidine thiocyanate were obtained from Sigma‐Aldrich, Zwijndrecht, the Netherlands while valsartan was from Novartis, Arnhem, the Netherlands. Angiotensinogen siRNA was provided by Alnylam Pharmaceuticals, Cambridge, MA, USA. Isoflurane was obtained from Zoetis, Rotterdam, the Netherlands. Paraformaldehyde was from Merck Life Science NV, Amsterdam, the Netherlands, and glutaraldehyde was from Aurion, Wageningen, the Netherlands.

### 2.9 Nomenclature of targets and ligands

Key protein targets and ligands in this article are hyperlinked to corresponding entries in the IUPHAR/BPS Guide to PHARMACOLOGY http://www.guidetopharmacology.org and are permanently archived in the Concise Guide to PHARMACOLOGY 2021/22 (Alexander, Christopoulos, et al., 2021; Alexander, Fabbro, et al., 2021).

## 3 RESULTS

### 3.1 Blood pressure lowering by angiotensinogen siRNA was identical to that by classical RAS blockers

**FIGURE 1:**
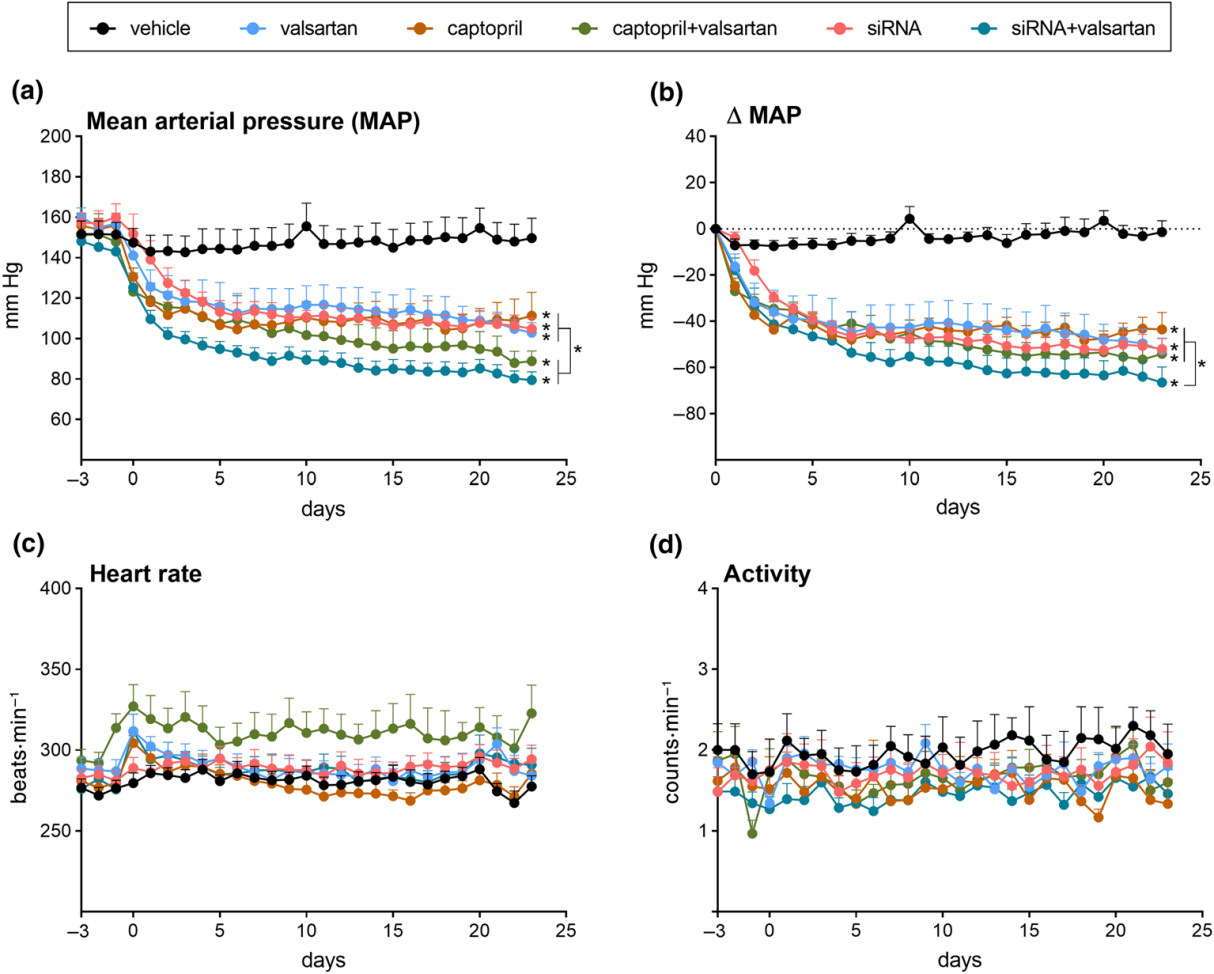
Effects on haemodynamics and activity. Mean arterial pressure (MAP; a), ΔMAP (b), heart rate (c) and locomotor activity (d) in diabetic Ren2 rats treated with either vehicle, valsartan, captopril, angiotensinogen (AGT) small interfering RNA (siRNA), AGT siRNA + valsartan, or captopril + valsartan for 3 weeks. Treatment was started 15 weeks after the induction of diabetes. Days −3 to 0 correspond to the period immediately before treatment. Data are mean ± SEM of n = 7–9. *P < 0.05 versus vehicle.

Mean arterial pressure (MAP) immediately prior to treatment was 153 ± 2 mmHg (n = 47). Vehicle did not alter MAP (Figure 1a). All treatments lowered MAP and the lowest MAP values were observed in the two dual treatment groups. However, when analysing ΔMAP, monotherapy with angiotensinogen siRNA, valsartan and captopril, and dual therapy with captopril + valsartan lowered MAP similarly by 52 ± 4, 52 ± 5, 43 ± 7 and 54 ± 6 mmHg, respectively, whereas the effect of dual therapy with angiotensinogen siRNA + valsartan (66 ± 6 mmHg) was larger than that of the other treatments (Figure 1b). Changes in systolic and diastolic blood pressure paralleled those in MAP and are shown in Figure S1. None of the treatments affected heart rate (Figure 1c) or locomotor activity (Figure 1d). MAP in four healthy Sprague–Dawley rats was 109 ± 1 mmHg.

### 3.2 Angiotensinogen siRNA lowered plasma angiotensinogen but left plasma angiotensin levels intact

Plasma angiotensinogen prior to diabetes induction amounted to 706 ± 32 pmol·ml^−1^ and plasma renin and prorenin were 41 ± 2 and 507 ± 33 ng angiotensin I per ml·h^−1^, respectively (Figure S2; n = 47 for all). At 15 weeks of diabetes, immediately prior to the treatment period, plasma renin was unchanged, prorenin had quadrupled to 1948 ± 179 ng angiotensin I per ml·h^−1^, and plasma angiotensinogen was reduced to 322 ± 22 pmol·ml^−1^ (Figure S2A–D). Given the much stronger correlation between prorenin and angiotensinogen versus that between renin and angiotensinogen (Figure S2E,F), the most likely reason for the drop in angiotensinogen is the rise in prorenin, considering that a small percentage of prorenin displays angiotensin I‐generating activity. (Krop et al., 2008).

**FIGURE 2:**
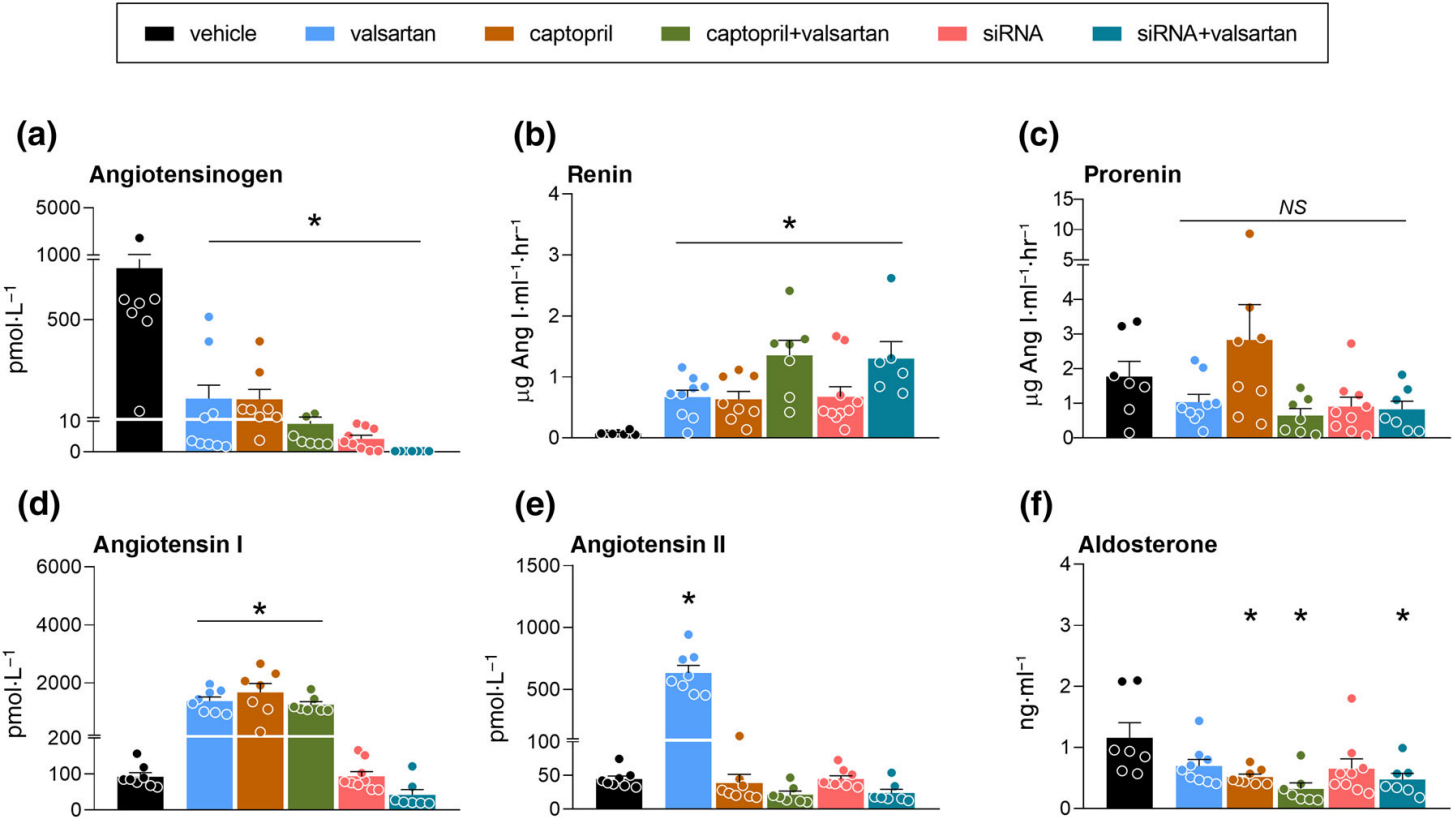
Effects on the circulating renin‐angiotensin system. Angiotensinogen (AGT; a), renin (b), prorenin (c), angiotensin I (Ang I; d), angiotensin II (e) and aldosterone (f) in blood plasma of diabetic Ren2 rats treated with either vehicle, valsartan, captopril, AGT small interfering RNA (siRNA), AGT siRNA + valsartan, or captopril + valsartan for 3 weeks. Treatment was started 15 weeks after the induction of diabetes. Data are mean ± SEM of n = 7–9. NS, not significant; *P < 0.05 versus vehicle.

Vehicle treatment did not alter angiotensinogen, renin or prorenin (Figures 2 and S2) in the diabetic animals. All RAS blockers lowered angiotensinogen (Figure 2a), but the greatest reductions were seen with angiotensinogen siRNA (by >99%) and angiotensinogen siRNA + valsartan (by 99.9%). Monotherapies increased renin by approximately 7‐fold (Figure 2b), whereas with both dual treatments, the increases were around 15‐fold (Figure 2b). None of the treatments affected prorenin (Figure 2c).

Valsartan, captopril and their combination increased plasma angiotensin I (Figure 2d and Table S2). Angiotensinogen siRNA did not alter plasma angiotensin I and although in combination with valsartan it resulted in the lowest plasma angiotensin I levels of all treatment groups, this was not significant versus vehicle (Figure 2d and Table S2). Valsartan increased plasma angiotensin II (Figure 2e and Table S2). None of the other treatments significantly altered plasma angiotensin II, although the lowest levels were observed in the dual treatment groups. Only captopril (alone and with valsartan) reduced the angiotensin II/I ratio (Table S2). The levels of angiotensin‐(2–8), angiotensin‐(3–8), angiotensin‐(1–7) and angiotensin‐(1–5) in the vehicle‐treated animals were around 1 order of magnitude lower than those of angiotensin II (Table S2). The treatment‐induced changes in angiotensin‐(2–8) and angiotensin‐(3–8) mimicked those in angiotensin II, whereas the changes in angiotensin‐(1–7) and angiotensin‐(1–5) mimicked those in angiotensin I (Table S2). Finally, captopril and the two dual treatments lowered aldosterone (Figure 2f and Table S2), whereas similar tendencies (NS) were observed for valsartan and angiotensinogen siRNA alone.

### 3.3 Angiotensinogen siRNA suppressed renal angiotensin levels without affecting renal angiotensinogen expression

**FIGURE 3:**
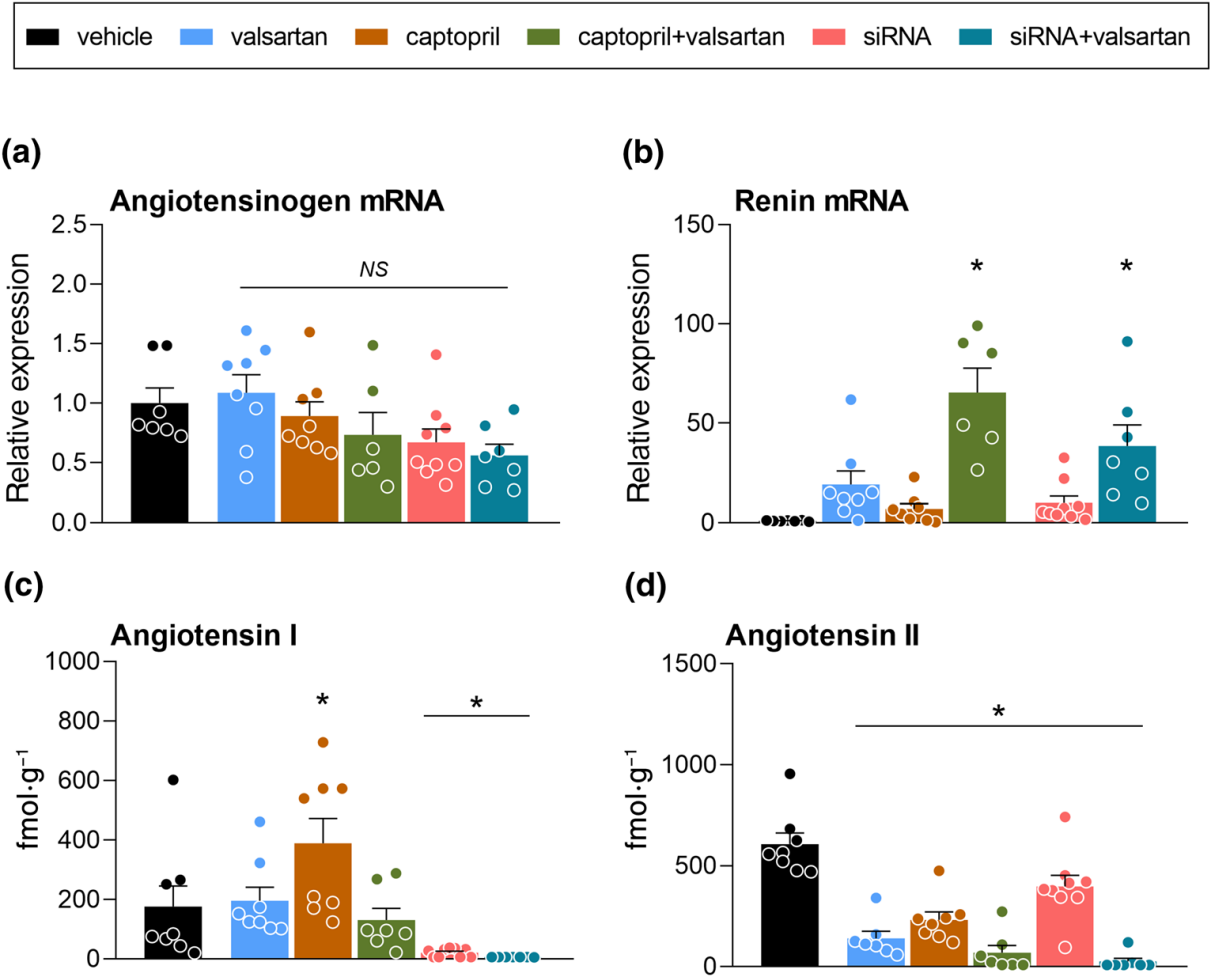
Effects on the renal renin‐angiotensin system. Expression of angiotensinogen (AGT; a) and renin (b), and tissue levels of angiotensin I (c) and angiotensin II (d) in the kidneys of diabetic Ren2 rats treated with either vehicle, valsartan, captopril, AGT small interfering RNA (siRNA), AGT siRNA + valsartan, or captopril + valsartan for 3 weeks. Treatment was started 15 weeks after the induction of diabetes. Data are mean ± SEM of n = 7–9. NS, not significant; *P < 0.05 versus vehicle.

No treatment affected renal angiotensinogen expression (Figure 3a). Angiotensinogen siRNA greatly lowered angiotensinogen protein in the kidney and this reduction was comparable with that in the liver (Figure S3). All treatments tended to up‐regulate renal renin mRNA, but significance was reached for angiotensinogen siRNA + valsartan (Figure 3b) and captopril + valsartan only. Captopril (Figure 3c and Table S2), but not valsartan, increased renal angiotensin I, whereas in combination with valsartan, captopril no longer increased renal angiotensin I. Angiotensinogen siRNA, alone or in combination with valsartan, virtually depleted renal angiotensin I. All treatments lowered renal angiotensin II (Figure 3d and Table S2), with angiotensinogen siRNA + valsartan almost completely abolishing renal angiotensin II levels. Valsartan, captopril and their combination lowered the angiotensin II/I ratio (Table S2). Finally, the renal levels of angiotensin‐(2–8), angiotensin‐(3–8), angiotensin‐(1–5) and angiotensin‐(1–7) after vehicle treatment were 1–2 orders of magnitude below those of angiotensin I and II, and largely unaffected by any of the inhibitors (Table S2). Captopril alone increased renal angiotensin‐(1–7) and valsartan alone increased renal angiotensin‐(1–5). No treatment altered the renal expression of the AT_1a_ or AT_1b_ receptor, nor that of Neph1, nephrin and podocin (Figure S4).

### 3.4 Angiotensinogen siRNA protected the diabetic kidney

**FIGURE 4:**
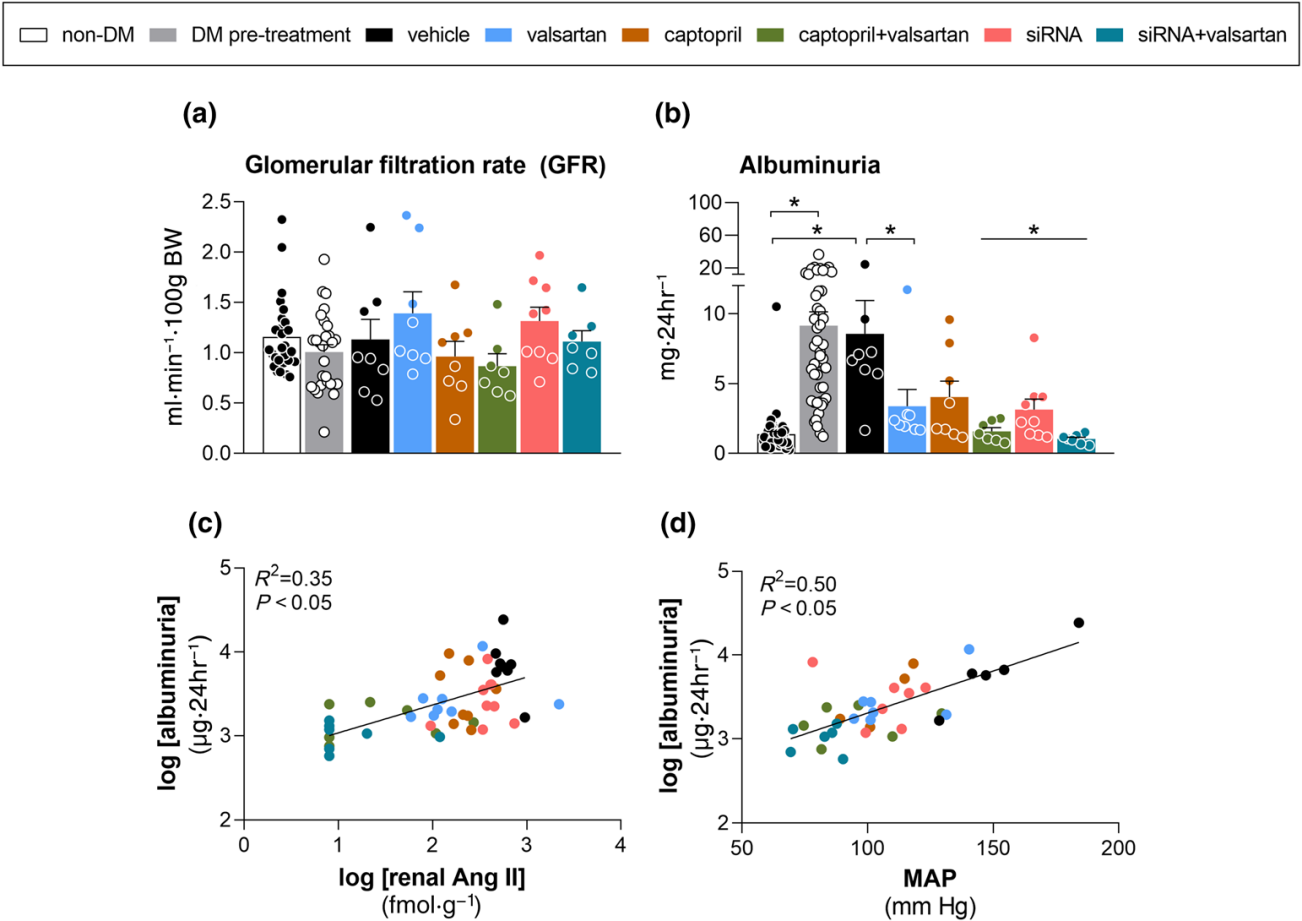
Effects on renal function. Glomerular filtration rate (GFR; a), albuminuria (b) and the correlation between albuminuria and renal angiotensin II (c) and mean arterial pressure (MAP), respectively in diabetic (DM) Ren2 rats treated with either vehicle, valsartan, captopril, angiotensinogen (AGT) small interfering RNA (siRNA), AGT siRNA + valsartan, or captopril + valsartan for 3 weeks. Treatment was started 15 weeks after the induction of diabetes. The first two columns in panels A and B depict time points before the induction of diabetes (white bar) and immediately before the start of treatment (grey bar) for all animals. Data are mean ± SEM of n = 7–9 (total n = 47). NS, not significant; *P < 0.05 versus vehicle or pre‐diabetes.

GFR at baseline amounted to 1.1 ± 0.06 ml·min^−1^ per 100 g of body weight (Figure 4a). Neither 15 weeks of diabetes nor any treatment altered GFR. As expected, diabetes greatly increased water intake and urine production (Table S3). None of the treatments altered this, nor did they alter sodium, potassium, urea, or creatinine excretion in the diabetic animals (Table S3). Captopril + valsartan increased plasma creatinine, plasma urea and serum potassium, whereas angiotensinogen siRNA + valsartan also increased plasma creatinine, but not plasma urea or serum potassium (Table S3). No other treatment affected these parameters, nor did any treatment affect serum sodium. Although the rise in plasma creatinine during dual treatment were accompanied by reductions in creatinine clearance, significance for these reductions was not reached.

Diabetes greatly increased albuminuria (Figure 4b) and all treatments prevented this, although the effect of captopril alone was of borderline significance (*P* = 0.06). Both dual treatments virtually normalized albuminuria. Renal angiotensin II and MAP both correlated strongly with albuminuria (Figure 4c,d). Incorporating MAP and renal angiotensin II in a multiple linear regression model revealed that MAP was the most important contributor (standardized coefficient MAP, 0.163; standardized coefficient renal angiotensin II, 0.008).

**FIGURE 5:**
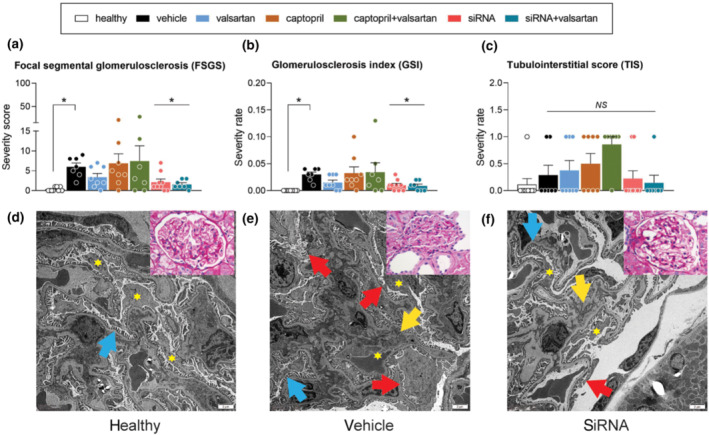
Renal histology. Panels A‐C. focal segmental glomerulosclerosis (FSGS; a), glomerulosclerosis index (GSI; b) and tubulointerstitial score (TIS; c) in kidneys of diabetic Ren2 rats treated with either vehicle, valsartan, captopril, angiotensinogen (AGT) small interfering RNA (siRNA), AGT siRNA + valsartan, or captopril + valsartan for 3 weeks. Treatment was started 15 weeks after the induction of diabetes. The first column depicts healthy Sprague–Dawley rats. Data are mean ± SEM of n = 7–9. NS, not significant; *P < 0.05 versus vehicle. Panels D–E. Electron microscopy pictures of glomeruli of healthy Sprague–Dawley rats (d), vehicle‐treated diabetic Ren2 rats (e) and AGT siRNA‐treated diabetic Ren2 rats (f). Stars depict podocytes, arrows indicate electron dense deposition in the mesangium (yellow), normal podocyte foot process (blue) and podocyte effacement (red). Inserts are periodic acid–Schiff‐stained renal histological images of glomeruli in the same animals.

The renal pathology scores focal segmental glomerulosclerosis and glomerulosclerosis index, but not tubulointerstitial score, followed a similar pattern as albuminuria (Figure 5a–c). Only angiotensinogen siRNA and angiotensinogen siRNA + valsartan reversed this outcome and a similar tendency (NS) was observed for valsartan. Electron microscopy revealed podocyte foot process effacement and electron dense mesangial deposition in the diabetic kidney (Figure 5e), which were not observed in healthy Sprague–Dawley rat kidneys (Figure 5d). Angiotensinogen siRNA partially prevented this outcome, with diminished podocyte foot processes effacement but unaltered electron dense deposition in the glomerular mesangium (Figure 5f).

### 3.5 Angiotensinogen siRNA protected the diabetic heart

**FIGURE 6:**
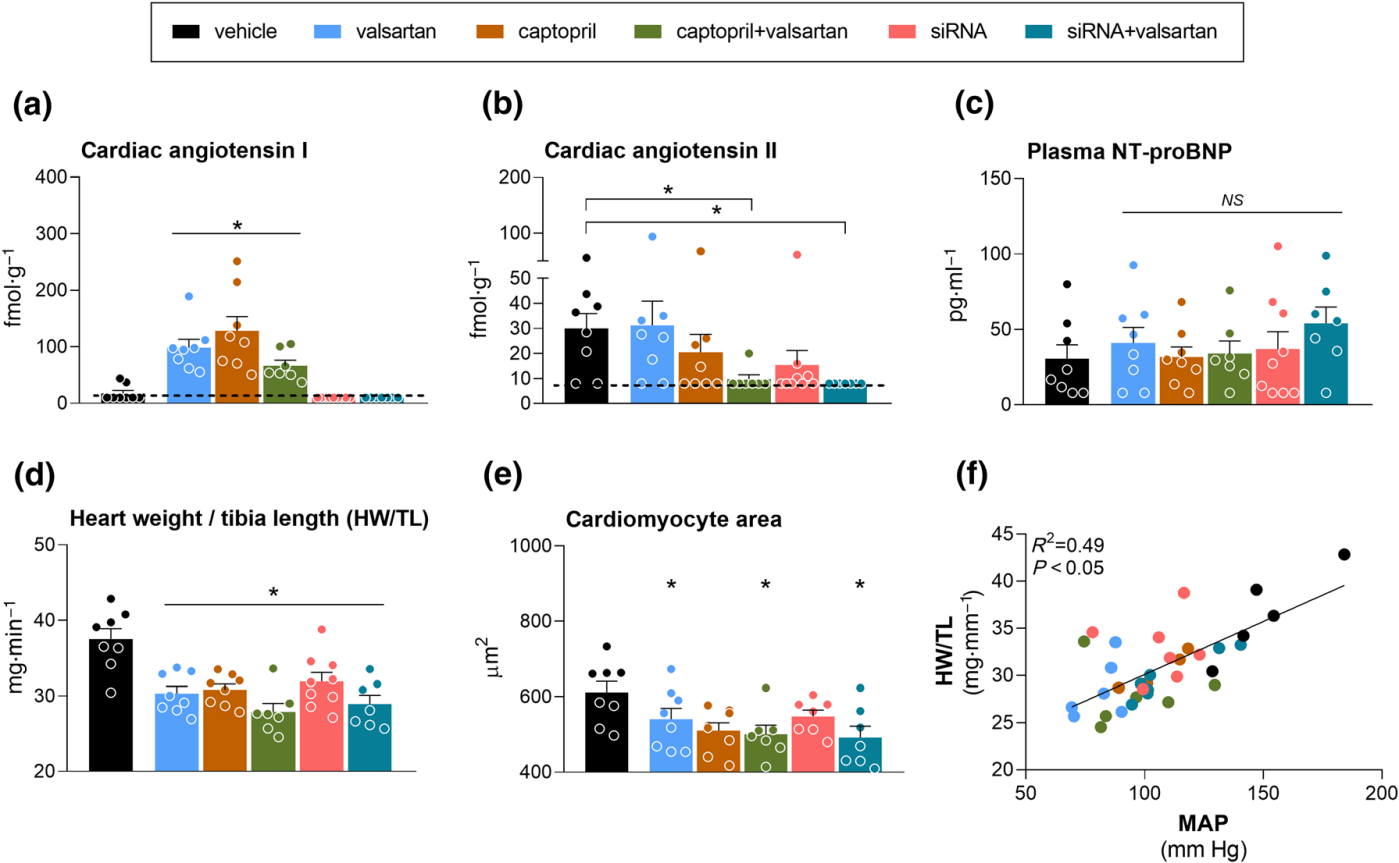
Effects on the heart. Cardiac angiotensin I (a) and angiotensin II (b) levels, plasma N‐terminal pro‐B‐type natriuretic peptide (NT‐proBNP) levels; c), heart weight/tibia length (HW/TL) ratio (d), cardiomyocyte area (e) and the relationship between HW/TL and MAP (f) in diabetic Ren2 rats treated with either vehicle, valsartan, captopril, angiotensinogen (AGT) small interfering RNA (siRNA), AGT siRNA + valsartan, or captopril + valsartan for 3 weeks. Treatment was started 15 weeks after the induction of diabetes. Data are mean ± SEM of n = 7–9. NS, not significant; *P < 0.05 versus vehicle.

Cardiac angiotensin I levels in vehicle‐treated rats were close to or below detection limit, whereas cardiac angiotensin II was easily detectable (Figures 6a,b). Valsartan, captopril or their combination increased cardiac angiotensin I, whereas during angiotensinogen siRNA treatment (alone or in combination with valsartan), cardiac angiotensin I remained undetectable. Cardiac angiotensin II was reduced only during blockade with angiotensinogen siRNA + valsartan and captopril + valsartan. Valsartan, captopril and their combination reduced the cardiac angiotensin II/I ratio (Table S2). Angiotensin‐(2–8), angiotensin‐(3–8), angiotensin‐(1–7) and angiotensin‐(1–5) were virtually undetectable during vehicle treatment and this was unaltered by RAS blocker treatment, except for a rise in cardiac angiotensin‐(1–7) after captopril and in angiotensin‐(1–5) after valsartan (Table S2). None of the treatments affected plasma N‐terminal pro‐B‐type natriuretic peptide (NT‐proBNP; Figure 6c). Yet all treatments reduced the heart weight/tibia length ratio versus the ratio in vehicle‐treated rats (Figure 6d) and a similar trend was observed for cardiomyocyte area (Figure 6e). The heart weight/tibia length ratio strongly correlated with MAP (Figure 6f). Cardiac fibrosis was barely detectable and no effects of treatment on this parameter could therefore be distinguished (data not shown).

### 3.6 Angiotensinogen siRNA and liver function

Diabetes increased both alanine aminotransferase (ALT) and aspartate aminotransferase (AST), but no treatment subsequently affected these parameters (Table S3).

## 4 DISCUSSION

This study demonstrates that a hepatocyte‐directed *N*‐acetylgalactosamine‐siRNA conjugate silencing the liver expression of angiotensinogen offers renoprotection in diabetic Ren2 rats. This effect involved both blood pressure lowering and angiotensin II suppression in the kidney, given that improved renal histology scores were seen with angiotensinogen siRNA alone and not with valsartan alone or captopril alone, despite the fact that all single treatments lowered blood pressure to the same degree. Moreover, renal angiotensin II levels were an independent determinant of albuminuria on top of blood pressure. In agreement with previous findings, all RAS blocker treatments additionally offered cardioprotection in this model.

The Ren2 rat, by overexpressing mouse (pro)renin, displays angiotensin II‐dependent hypertension (Mullins et al., 1990). In this model, diabetes induction increased prorenin rather than renin (Figure S2), in agreement with observations in humans that diabetes is characterized by high prorenin levels and low‐to‐normal renin levels. Prorenin is the inactive precursor of renin. This inactivity is due to the fact that the so‐called prosegment covers renin's active site. Nevertheless, in a small percentage of the prorenin molecules (around 1%–2% under physiological conditions), the prosegment may move away from the active site, thus allowing prorenin to display some activity (Krop et al., 2013). As a consequence, particularly when prorenin levels are several orders of magnitude above those of renin (like in this model), prorenin may contribute to angiotensin generation. This explains why we observed a strong negative association between prorenin and angiotensinogen and lower angiotensinogen levels following the induction of diabetes in the Ren2 rat.

All RAS blockers further lowered circulating angiotensinogen in the diabetic animals. In the case of captopril and valsartan this was due to the fact that these drugs interfere with angiotensin II synthesis or its AT_1_ receptor‐mediated effects, thereby blocking the negative feedback loop between angiotensin II and renin release. This results in renin rises, which obviously cause angiotensinogen depletion. In the case of angiotensinogen siRNA, the lowering is due to direct blockade of its synthesis in the liver. This effect is highly effective, as evidenced by the >99% suppression of circulating and hepatic angiotensinogen after single angiotensinogen siRNA treatment. As a consequence, the circulating angiotensinogen levels in the rat, which normally resemble those in humans, become as low as those in mice. Mice display much higher renin levels than humans (Fraune et al., 2012; van Thiel et al., 2017), thereby allowing them to still display the same circulating angiotensin levels as humans and rats, despite their low angiotensinogen levels. Our data in Ren2 rats reveal that angiotensinogen suppression results in a renin rise that is sufficiently high to keep the circulating angiotensin levels in the normal range. Combining angiotensinogen siRNA with valsartan lowered circulating angiotensinogen nearly completely (by 99.9%), most likely because this approach increased renin even further. Nevertheless, also under those circumstances, circulating angiotensin II levels remained in the (low) normal range. This demonstrates how capable the RAS is of keeping circulating angiotensin II in a desired range. Indeed, in humans renin rises of many hundred‐fold are not uncommon, for example, during renin inhibition (Balcarek et al., 2014), to prevent the entire disappearance of circulating angiotensin II. Yet RAS blockade did substantially lower blood pressure in the Ren2 rat and this therefore most likely represents interference with tissue angiotensin II, for instance in the kidney (see below). The low aldosterone levels during most treatments support that this also occurred at the level of the adrenal. Unlike the spontaneously hypertensive rat, where dual RAS blockade with either angiotensinogen siRNA + valsartan or captopril + valsartan synergistically lowered blood pressure (Uijl et al., 2019), in the Ren2 rat, the antihypertensive effect of dual blockade with captopril + valsartan was identical to that of each drug alone, whereas the effect of angiotensinogen siRNA + valsartan was marginally (albeit significantly) larger than that of each drug alone. This suggests that the high blood pressure in this model is largely the consequence of Ren2 overexpression and not related to diabetes per se. In other words, as soon as blood pressure is back to normal (by blocking the effects of Ren2 overexpression), more RAS blockade does not yield substantial further blood pressure lowering.

The pattern of RAS suppression in the kidney following the various treatments was entirely different from that in the circulation. All treatments lowered renal angiotensin II, yet without affecting renal angiotensinogen expression. The latter is in agreement with all previous studies in rat models with angiotensinogen siRNA (Bovée et al., 2021; Kukida et al., 2021; Uijl et al., 2019, 2021) and supports the liver‐specificity of this approach. Yet the angiotensinogen protein disappeared from renal tissue sites after angiotensinogen siRNA treatment. Taken together, these data support that the angiotensinogen protein that contributes to angiotensin II generation at renal tissue sites is liver‐derived. Indeed, combining angiotensinogen siRNA + valsartan almost annihilated renal angiotensin II, independent of any change in the renal angiotensinogen mRNA levels. Recent data suggest that the uptake of hepatic angiotensinogen in the kidney involves megalin (Koizumi et al., 2019; Sun et al., 2020; Ye et al., 2018). Angiotensinogen siRNA also substantially down‐regulated the renal angiotensin I levels, suggesting that in the absence of liver angiotensinogen, local angiotensin I generation at renal tissue sites is no longer possible. No such angiotensin I decrease was seen with either captopril or valsartan (in fact, during captopril, renal angiotensin I levels even increased) and thus the drop in renal angiotensin II levels reflects either ACE inhibition or diminished angiotensin II binding to AT_1_ receptors with valsartan. These data therefore argue against a role for non‐ACE converting enzymes (like chymase) and simultaneously agree with the earlier observation that tissue angiotensin II is largely, if not completely AT_1_ receptor‐bound (van Esch, Gembardt, et al., 2010). No drug affected AT_1_ receptor expression and thus diminished angiotensin II‐AT_1_ receptor interaction was not counterbalanced by a larger density of AT_1_ receptors. Importantly, when focusing on other angiotensin metabolites, we observed that both angiotensin III and angiotensin‐(1–7) were present in renal tissue, albeit at substantially lower levels than angiotensin I and II. Their levels remained low or became undetectable, during all treatments, with the exception of angiotensin‐(1–7) which rose in parallel with angiotensin I during ACE inhibition. This most likely reflects the shift from angiotensin I‐II conversion by ACE to angiotensin I‐angiotensin‐(1–7) conversion by neutral endopeptidase (Kaltenecker et al., 2020). Nevertheless, no apparent additional positive consequences were observed from this renal angiotensin‐(1–7) up‐regulation during ACE inhibition. Taken together, our data suggest that the renoprotective effects of all RAS blockers reflect renal angiotensin II blockade and such selective tissue angiotensin II suppression most likely also underlies the antihypertensive effects of these drugs.

The renoprotective effects concerned a reduction or even normalization of albuminuria in the diabetic animals. Diabetes had not yet affected GFR in our model and thus no improvement in GFR was to be expected. Albuminuria correlated with both MAP and renal angiotensin II, and when incorporating both parameters in a multiple linear regression model, MAP appeared to be the most important contributor. Like in earlier studies, we were unable to demonstrate beneficial effects of monotherapy with an AT_1_ antagonist or ACE inhibitor on glomerulosclerosis (Mifsud et al., 2002; Roksnoer et al., 2016; Uijl et al., 2020), nor of their combined treatment. Only angiotensinogen siRNA, with or without valsartan, prevented this and electron microscopy revealed that such treatment involved partial normalization of the podocyte foot process effacement and electron dense mesangial deposition in the diabetic kidney. These beneficial effects occurred independently of changes in the expression levels of the podocyte markers Neph1, nephrin and podocin, and in case of angiotensinogen siRNA monotherapy, at the same degree of blood pressure lowering as observed during AT_1_ antagonist or ACE inhibitor monotherapy. Taken together, improved renal pathology apparently is not only possible by combining a classical RAS blocker with a neprilysin inhibitor, as we have demonstrated before (Uijl et al., 2020), but also by applying angiotensinogen siRNA as a single treatment, presumably because it effectively reduces angiotensin II at the level of the glomerulus. It seems reasonable to assume that such effects can also be achieved with higher doses of either captopril or valsartan alone, but possibly at the cost of more side effects.

All treatments exerted an identical degree of protection in the diabetic heart, reflected by similar reductions in cardiac hypertrophy and cardiomyocyte area. This occurred despite the absence of significant reductions in cardiac angiotensin II and therefore most likely reflects the consequences of blood pressure lowering. Indeed, MAP correlated strongly with the heart weight/tibia length ratio, although no changes in N‐ternimal pro‐B‐type natriuretic peptide levels were observed following treatment.

It has been argued that liver‐targeting of angiotensinogen siRNA may exert liver toxicity, or inflammatory and immunological side effects. Yet no such observations were made for liver‐targeted proprotein convertase subtilisin/kexin type 9 siRNA (inclisiran) over a 6‐month period (Landmesser et al., 2021). Indeed, with a number of approved clinical products and with a substantial body of preclinical investigative work (Janas et al., 2019; Janas, Harbison, et al., 2018; Janas, Schlegel, et al., 2018), it is now well established that liver‐targeting *N*‐acetylgalactosamine‐siRNA conjugates have favourable preclinical safety profiles and wide therapeutic windows. In line with this, our data on the liver enzymes alanine aminotransferase and aspartate aminotransferase in diabetic Ren2 rats do not show any liver deterioration after angiotensinogen siRNA treatment, either alone or in combination with valsartan. Nevertheless, future studies, for instance making use of scrambled siRNA, are warranted to ascertain that no off‐target effects occur with this approach.

Finally, the well‐known consequences of too much RAS blockade in humans are renal dysfunction and hyperkalaemia (Danser & van den Meiracker, 2015; Parving et al., 2012). The former tended to occur during both dual RAS blocker treatments and the latter did occur during treatment with valsartan + captopril. However, neither of these safety signals was observed with single siRNA treatment. Additionally, serum potassium and urea were normal with siRNA + valsartan but elevated with valsartan + captopril, further supporting the potential for an improved safety profile by targeting liver production of angiotensinogen + AT_1_ antagonist compared with dual RAS blockade. Obviously, the next step will be to establish what degree of RAS blockade is optimal when involving angiotensinogen suppression. Here it is important to note that there may be emergency situations where the RAS is acutely needed, like shock and other conditions that cause hypotension. In the absence of sufficient angiotensinogen, rapid RAS activation would not be possible. We have mimicked this situation by treating spontaneously hypertensive rats on a low‐salt diet with angiotensinogen siRNA (Uijl et al., 2022). The drop in blood pressure could be counteracted acutely by infusing the vasopressors noradrenaline or angiotensin II, and chronically by vasopressor‐sparing strategies such as high salt and fludrocortisone. Thus, there is a pharmacological escape to raise arterial pressure and maintain perfusion pressure when angiotensinogen levels are low.

In summary, a liver‐targeted *N*‐acetylgalactosamine‐siRNA conjugate that silences the hepatic expression of angiotensinogen exerted reno‐ and cardioprotection in diabetic Ren2 rats. These effects reflect the dependency of both renal and cardiac angiotensin II on liver‐derived angiotensinogen. Angiotensinogen siRNA seems to be the first type of single RAS blockade that not only prevented albuminuria, but also improved renal pathology in this model. In view of the potential long‐lasting effects of silencing with *N*‐acetylgalactosamine‐siRNA conjugates treatment (such as with inclisiran, which is dosed every 6 months (Ray et al., 2017) targeting hepatic angiotensinogen offers new possibilities for the treatment of CKD in diabetic patients. Future studies are needed to understand both the long‐term risks and benefits of such long‐term tissue RAS suppression, considering that too much renal angiotensin II suppression may result in renal dysfunction and hyperkalaemia. Such studies should include both alternative (non‐Ren2) diabetes models without hypertension, as well as clinical trials with this novel approach.

## AUTHOR CONTRIBUTIONS

**Edwyn O. Cruz‐López:** Conceptualization; data curation; formal analysis; investigation; methodology; project administration. **Liwei Ren:** Data curation; formal analysis; investigation; methodology; project administration. **Estrellita Uijl:** Conceptualization; formal analysis; methodology; supervision. **Marian C. Clahsen‐van Groningen:** Data curation; formal analysis; methodology. **Richard van Veghel:** Data curation; investigation; methodology. **Ingrid M. Garrelds:** Data curation; investigation; methodology. **Oliver Domenig:** Data curation; investigation; methodology. **Marko Poglitsch:** Formal analysis; investigation; methodology. **Timothy Rooney:** Conceptualization. **Anne Kasper:** Conceptualization. **Paul Nioi:** Conceptualization. **Don Foster:** Conceptualization.

## CONFLICTS OF INTEREST

I. Zlatev, T. Rooney, A. Kasper, P. Nioi, D. Foster are employees of Alnylam Pharmaceuticals. A.H.J. Danser received a grant from Alnylam Pharmaceuticals, which has partially supported this work. O. Domenig and M. Poglitsch are employees of Attoquant Diagnostics. The other authors report no conflicts.

## DECLARATION OF TRANSPARENCY AND SCIENTIFIC RIGOUR

This Declaration acknowledges that this paper adheres to the principles for transparent reporting and scientific rigour of preclinical research as stated in the *BJP* guidelines for Design & Analysis, Immunoblotting and Immunochemistry and Animal Experimentation, and as recommended by funding agencies, publishers and other organizations engaged with supporting research.

## Supporting information


**Table S1.** List of qPCR primer sequences.
**Table S2.** Angiotensin metabolites in blood, kidney and heart of diabetic Ren2 rats treated with either vehicle, valsartan, captopril, angiotensinogen (AGT) small interfering RNA (siRNA), AGT siRNA + valsartan, or captopril + valsartan for 3 weeks. Data are mean±SD of n = 7–9. Numbers in combination with the symbol < denote the lower limit of quantification. *P < 0.05 versus vehicle.
**Table S3.** Main characteristics of diabetic Ren2 rats treated with either vehicle, valsartan, captopril, angiotensinogen (AGT) small interfering RNA (siRNA), AGT siRNA + valsartan, or captopril + valsartan for 3 weeks. Data prior to the induction of diabetes mellitus (DM) and treatment are also provided. Data are mean±SD. #*P* < 0.05 versus non‐DM; *P < 0.05 versus vehicle.
**Figure S1.** Systolic and diastolic blood pressure (SBP, DBP) in diabetic Ren2 rats treated with either vehicle, valsartan, captopril, angiotensinogen small interfering RNA (siRNA), AGT siRNA + valsartan, or captopril + valsartan for 3 weeks. Treatment was started 15 weeks after the induction of diabetes. Days −3 to 0 correspond to the period immediately before treatment. Data are mean±SEM of n = 7–9. *P <0 .05 versus vehicle and/or indicated group.
**Figure S2.** Effect of diabetes induction on the levels of angiotensinogen (A), renin, (B) and renin (C) in Ren2 rats after 15 weeks and the relationship between renin (D) or prorenin (E) and angiotensinogen. Data are mean±SEM of n = 47; *P < 0.05.
**Figure S3.** Hepatic (panel A) and renal (panel B) angiotensinogen levels in diabetic Ren2 rats treated with either vehicle, angiotensinogen (AGT) small interfering RNA (siRNA) or AGT siRNA + valsartan. Treatment was started 15 weeks after the induction of diabetes. Panel C shows the immunoblots. Data are mean±SEM of n = 7–9. *P < 0.05 versus vehicle.
**Figure S4.** Renal expression of the angiotensin II type 1a and 1b (AT1a, AT1b) receptor (A and B), Neph1 (C), nephrin (D) and podocin (E) in diabetic Ren2 rats treated with either vehicle, valsartan, captopril, angiotensinogen (AGT) small interfering RNA (siRNA), AGT siRNA + valsartan, or captopril + valsartan for 3 weeks. Treatment was started 15 weeks after the induction of diabetes. Data are mean±SEM of n = 7–9. NS, not significant.

## Data Availability

All supporting data are available within the article and in the supporting information.
