# Supplementary material for: Blood pressure‐independent renoprotective effects of small interference RNA targeting liver angiotensinogen in experimental diabetes
Source: Br J Pharmacol. 2022 Oct 2;180(1):80–93. doi: 10.1111/bph.15955 (PMC10091936; doi:10.1111/bph.15955)

Blood pressure-independent renoprotective effects of small interference RNA targeting liver angiotensinogen in experimental diabetes

Edwyn O. Cruz-López[1]*, Liwei Ren[1,2]*, Estrellita Uijl[1,3], Marian C. Clahsen-van Groningen[4,5], Richard van Veghel[1], Ingrid M. Garrelds[1], Oliver Domenig[6], Marko Poglitsch[6], Ivan Zlatev[7], Timothy Rooney[7], Anne Kasper[7], Paul Nioi[7], Don Foster[7], A.H. Jan Danser[1]

[1]Division of Vascular Medicine and Pharmacology, Department of Internal Medicine, Erasmus MC, Rotterdam, The Netherlands; [2]Department of Pharmacy, Shenzhen People's Hospital (The Second Clinical Medical College, Jinan University, The First Affiliated Hospital, Southern University of Science and Technology), Shenzhen, China; [3]Division of Nephrology and Transplantation, Department of Internal Medicine, Erasmus MC, Rotterdam, The Netherlands; [4]Department of Pathology, Erasmus MC, Rotterdam, The Netherlands; [5]Institute of Experimental Medicine and Systems Biology, University Hospital Aachen, RWTH Aachen University, Aachen, German; [6]Attoquant Diagnostics, Vienna, Austria; [7]Alnylam Pharmaceuticals, Cambridge, MA, USA.

**both authors contributed equally.*

Correspondence: Prof. dr. A. H. Jan Danser, PhD; Division of Pharmacology, room EE1418b, Erasmus MC, Wytemaweg 80, 3015 CN Rotterdam, The Netherlands (e-mail: a.danser@erasmusmc.nl).

SUPPLEMENTAL MATERIAL

Table S1. List of qPCR primer sequences.

**Gene**		**Sequence (5' to 3')**
Agt	Forward primer	CCAGCACGACTTCCTGACTT
	Reverse primer	GCAGGTTGTAGGATCCCCGA
Renin	Forward primer	TGTGGTAACTGTGGGTGGAAT
	Reverse primer	GCATGAAGGGTATCAGGGGC
AT_1aR	Forward primer	ATCACCAGGTCAAGTGGATTTCG
	Reverse primer	TTCCCACCACAAAGATGATGC
AT_1bR	Forward primer	CTGAATCTTGCCCTGGCTGA
	Reverse primer	ACATAGGTGGTTGCCGAAGG
B2M	Forward primer	ATGGCTCGCTCGGTGACCG
	Reverse primer	TGGGGAGTTTTCTGAATGGCAAGCA
Actb	Forward primer	GGGAAATCGTGCGTGACATT
	Reverse primer	GCGGCAGTGGCCATCTC
Neph1	Forward primer	GGATGGCGGTAAGGTGGAGTG
	Reverse primer	CGTTATTGATGGTGAGAGTGGACAG
Nephrin	Forward primer	CGTCAGCATCAGCAGCAACC
	Reverse primer	AGCCACATCTTCCAGCCTCTC
Podocin	Forward primer	TGGACTCAGTGACCTGTGTTTGG
	Reverse primer	CAGCAATCACCCGCACTTTGG
WT-1	Forward primer	TGTGACTTCAAGGACTGCGAGAG
	Reverse primer	GGTGTGGGTCTTCAGGTGGTC

Agt, angiotensinogen; $AT_{1a}R$, angiotensin II type 1a receptor; $AT_{1b}R$, angiotensin II type 1b receptor; B2M, β_2-microglobulin; Actb, β-actin; WT-1, Wilms tumor-1.

Table S2. Angiotensin metabolites in blood, kidney and heart of diabetic Ren2 rats treated with either vehicle, valsartan, captopril, angiotensinogen (AGT) small interfering RNA (siRNA), AGT siRNA + valsartan, or captopril + valsartan for 3 weeks. Data are mean±SD of n=7-9. Numbers in combination with the symbol < denote the lower limit of quantification. *P<0.05 versus vehicle.

Angiotensin metabolite	vehicle	valsartan	captopril	captopril+ valsartan	siRNA	siRNA+ valsartan
Blood (pmol/L)						
**Ang-(1-10) = Ang I**	93±31	1,378±389*	1,675±806*	1,251±272*	94±40	42±39
**Ang-(1-8) = Ang II**	45±13	633±170*	39±35	22±13	45±13	24±15
**Ang-(1-7)**	8.2±0.6	38±19*	80±42*	44±5.7*	10±6.0	<8
**Ang-(2-8) = Ang III**	4.5±2.8	86±34*	8.0±11	<2.5	3.9±1.2	2.9±1.2
**Ang-(3-8) = Ang IV**	3.5±1.7	59±21*	6.4±8.8	2.3±0.6	3.9±1.7	2.1±0.3
**Ang-(1-5)**	10.4±3.0	136±59*	8.7±5.1	6.4±2.4	8.0±2.4	7.2±6.3
**Ang II/I ratio**	0.5±0.1	0.5±0.0	0.0±0.0*	0.0±0.0*	0.5±0.1	0.7±0.1*
Kidney (fmol/g)						
**Ang-(1-10) = Ang I**	176±195	195±128	388±237*	131±103	21±14*	<6*
**Ang-(1-8) = Ang II**	606±158	140±93*	232±109*	68±97*	396±165*	26±42*
**Ang-(1-7)**	32±28	92±101	195±123*	51±50	23±11	<15
**Ang-(2-8) = Ang III**	41±22	24±48	33±35	10±12	25±12	<6
**Ang-(3-8) = Ang IV**	<10	<10	<10	<10	<10	<10
**Ang-(1-5)**	12±6.1	58±39*	7.5±4.3	7.0±4.0	6.0±2.0	<5
**Ang II/I ratio**	8.0±2.5	1.0±0.3	0.9±0.2	0.5±0.2	36±13*	4.3±2.6
Heart (fmol/g)						
**Ang-(1-10) = Ang I**	18±14	99±42*	128±71*	67±26*	<10	<10
**Ang-(1-8) = Ang II**	30±17	31±27	20±20	9.7±4.5*	15±17	<8*
**Ang-(1-7)**	<15	25±28	55±56*	<15	<15	<15
**Ang-(2-8) = Ang III**	<10	<10	<10	<10	<10	<10
**Ang-(3-8) = Ang IV**	<10	<10	<10	<10	<10	<10
**Ang-(1-5)**	5.4±1.2	65±22*	7.8±5.3	6.0±2.7	5.1±0.3	<5
**Ang II/I ratio**	2.3±1.6	0.3±0.2*	0.1±0.1*	0.2±0.0*	1.5±1.7	0.8±0.0*

Table S3. Main characteristics of diabetic Ren2 rats treated with either vehicle, valsartan, captopril, angiotensinogen (AGT) small interfering RNA (siRNA), AGT siRNA + valsartan, or captopril + valsartan for 3 weeks. Data prior to the induction of diabetes mellitus (DM) and treatment are also provided. Data are mean±SD. $^{\#}$P<0.001 versus non-DM; *P<0.05 versus vehicle).

Parameters	0 weeks	15 weeks	18 weeks					
	non-DM	DM	vehicle	valsartan	captopril	captopril +valsartan	AGT siRNA	AGT siRNA +valsartan
N	47	47	8	8	8	7	9	7
**General**								
body weight (g)	395±38	401±44	448±37	435±29	411±31	388±15*	422±44	416±46
Δtreatment (g)			26±21	31±28	19±18	24±12	9.6±17	12±34
food intake (g/day)	25±6	35±12$^{\#}$	25±10	28±8.3	38±6.7*	28±11	30±8.1	29±5.5
water intake (mL/day)	38±8	136±52$^{\#}$	119±55	123±56	173±58	133±67	150±76	113±59
serum glucose (mmol/L)			39±11	38±21	52±14	55±10	46±6	50±13
insulin (U/day)			3.6±1.3	3.5±1.1	3.3±1.2	3.0±1.1	3.0±1.1	3.6±1.4
**Blood**								
plasma ALT (U/L)	56±14	181±130$^{\#}$	239±122	181±213	117±38	116±100	145±43	116±54
plasma AST (U/L)	85±30	280±267$^{\#}$	327±204	265±324	150±64	233±201	186±95	179±167
serum K^+ (mmol/L)			6.3±1.8	6.9±1.3	8.2±1.6	9.0±1.0*	7.2±2.3	7.7±1.3
serum Na^+ (mmol/L)			134±4.8	134±6.5	128±4.3	128±4.2	130±4.1	128±4.7
plasma urea (mmol/L)	5.1±0.9	8.9±1.6$^{\#}$	6.6±1.1	7.4±1.6	8.5±1.1	21±14*	8.7±1.5	14±4.5
plasma creatinine (µmol/L)	25±5	19±4$^{\#}$	19±2.1	23±4.8	19±3.8	41±24*	24±9.2	37±14*
**Urine**								
urine volume (mL/day)	22±6	132±53$^{\#}$	128±48	126±65	155±50	109±44	134±64	104±57
K^+ excretion (mmol/day)	3.1±0.6	4.8±1.2$^{\#}$	3.5±1.7	4.3±1.6	4.6±1.4	3.0±0.9	3.7±1.2	3.9±1.6
Na^+ excretion (mmol/day)	2.3±0.6	3.3±1.1$^{\#}$	2.5±1.4	2.8±1.3	3.3±1.0	2.1±0.6	2.3±1.1	2.3±0.7
urea excretion (mmol/day)	12±2.0	23±5.2$^{\#}$	15±8.2	20±8.4	20±6.7	13±3.7	18±5.1	17±7.9
creatinine excretion (µmol/day)	128±20	123±27	82±44	120±35	87±31	74±28	96±33	111±38
creatinine clearance (mL/min)	3.8±0.9	4.6±1.2$^{\#}$	3.2±2.1	3.6±1.1	3.2±1.0	1.4±0.6	2.9±1.0	2.6±1.5

Figure S1. Systolic and diastolic blood pressure (SBP, DBP) in diabetic Ren2 rats treated with either vehicle, valsartan, captopril, angiotensinogen (AGT) small interfering RNA (siRNA), AGT siRNA + valsartan, or captopril + valsartan for 3 weeks. Treatment was started 15 weeks after the induction of diabetes. Days -3 to 0 correspond to the period immediately before treatment. Data are mean±SEM of n=7-9. *P<0.05 versus vehicle and/or indicated group.

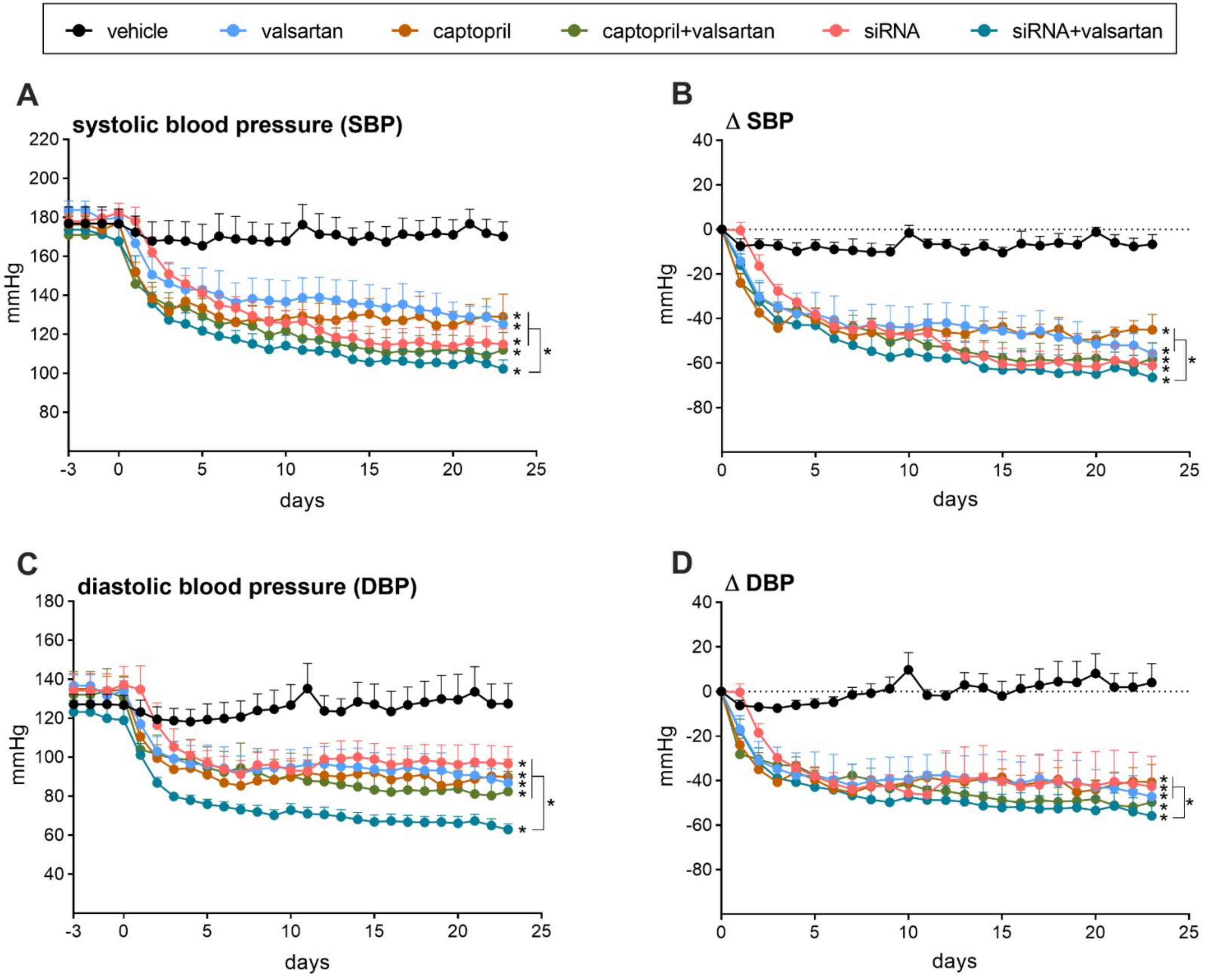

Figure S2. Effect of diabetes induction on the levels of angiotensinogen (A), renin, (B), and renin (C) in Ren2 rats after 15 weeks, and the relationship between renin (D) or prorenin (E) and angiotensinogen. Data are mean±SEM of n=47; *P<0.0001.

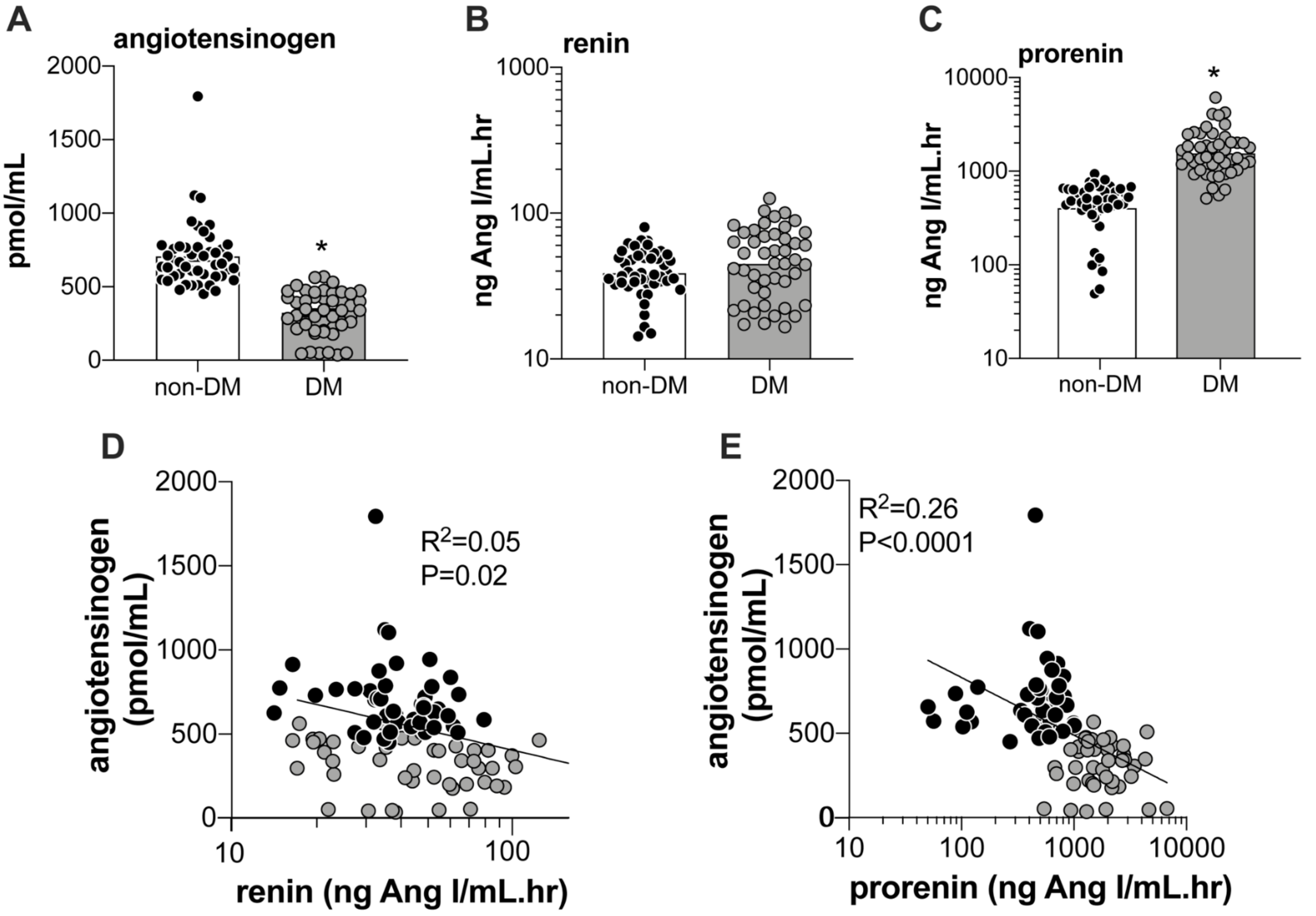

Figure S3. Hepatic (panel A) and renal (panel B) angiotensinogen levels in diabetic Ren2 rats treated with either vehicle, angiotensinogen (AGT) small interfering RNA (siRNA) or AGT siRNA + valsartan**.** Treatment was started 15 weeks after the induction of diabetes. Panel C shows the immunoblots. Data are mean±SEM of n=7-9. *P<0.05 versus vehicle.

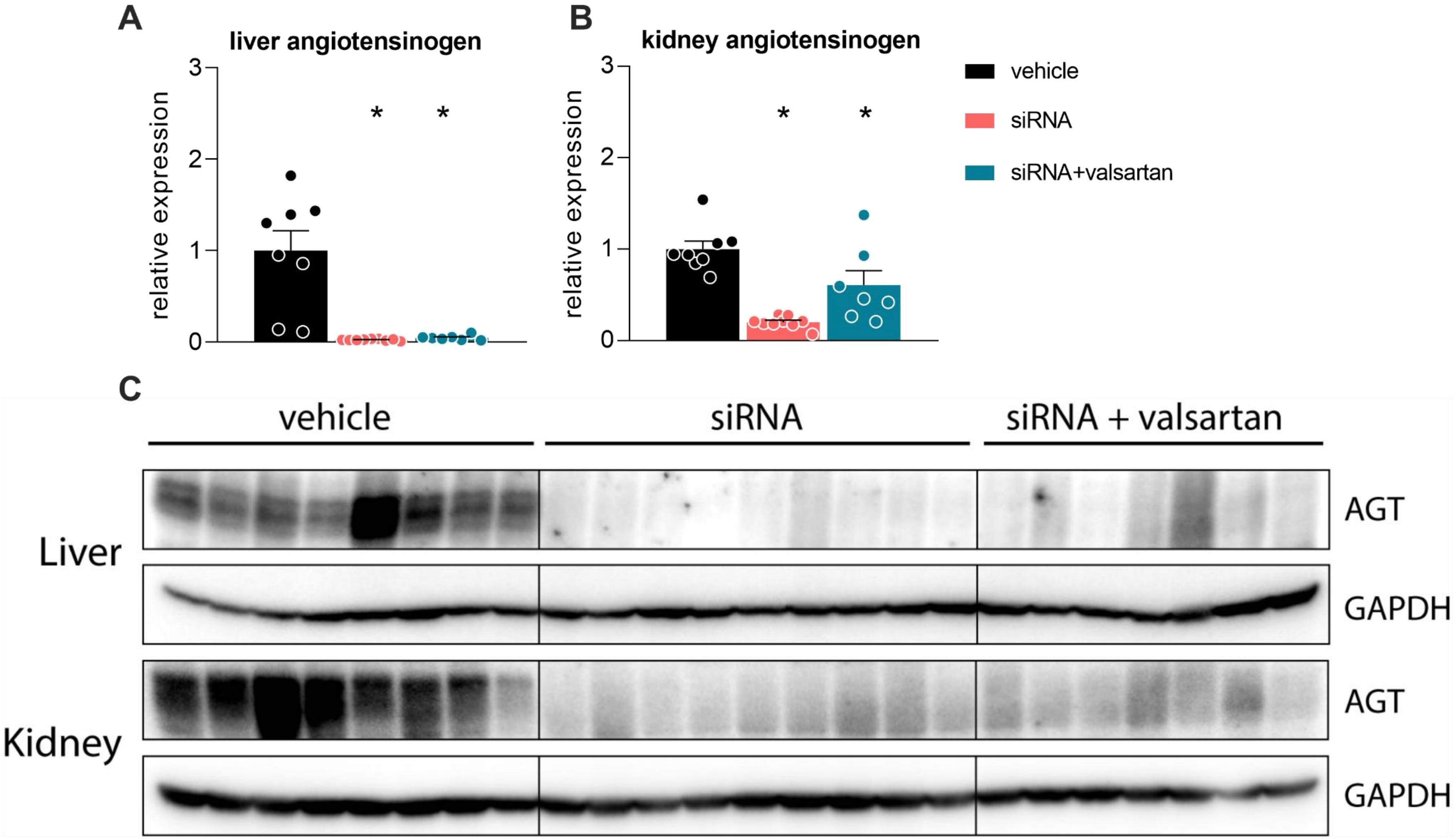

Figure S4. Renal expression of the angiotensin II type 1a and 1b (AT_{1a}, AT_{1b}) receptor (A and B), Neph1 (C), nephrin (D) and podocin (E) in diabetic Ren2 rats treated with either vehicle, valsartan, captopril, angiotensinogen (AGT) small interfering RNA (siRNA), AGT siRNA + valsartan, or captopril + valsartan for 3 weeks. Treatment was started 15 weeks after the induction of diabetes. Data are mean±SEM of n=7-9. NS, not significant.

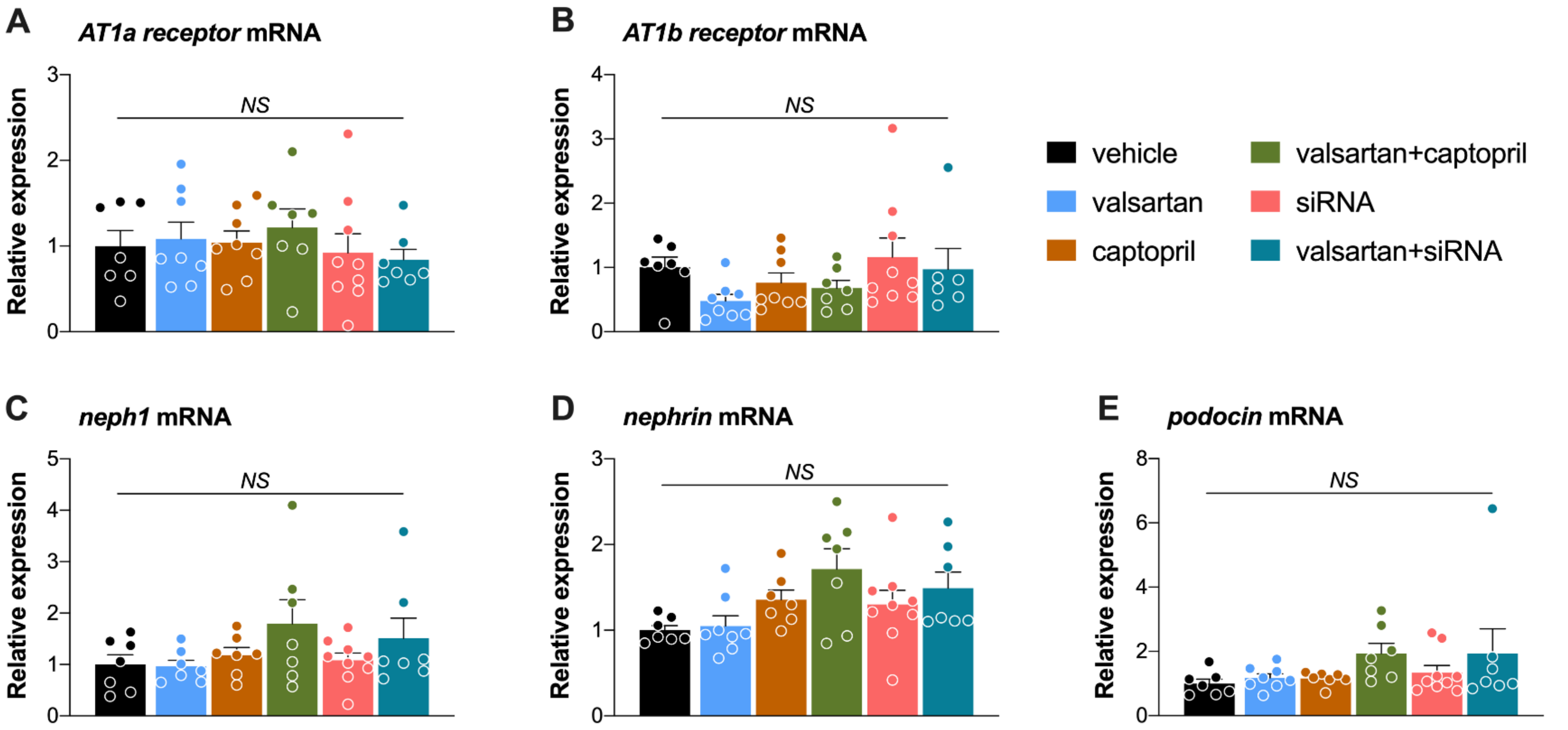

Supplement: Supplementary file 1 — Table S1. List of qPCR primer sequences. Table S2. Angiotensin metabolites in blood, kidney and heart of diabetic Ren2 rats treated with either vehicle, valsartan, captopril, angiotensinogen (AGT) small interfering RNA (siRNA), AGT siRNA + valsartan, or captopril + valsartan for 3 weeks. Data are mean±SD of n = 7–9. Numbers in combination with the symbol < denote the lower limit of quantification. *P < 0.05 versus vehicle. Table S3. Main characteristics of diabetic Ren2 rats treated with either vehicle, valsartan, captopril, angiotensinogen (AGT) small interfering RNA (siRNA), AGT siRNA + valsartan, or captopril + valsartan for 3 weeks. Data prior to the induction of diabetes mellitus (DM) and treatment are also provided. Data are mean±SD. #P < 0.05 versus non‐DM; *P < 0.05 versus vehicle. Figure S1. Systolic and diastolic blood pressure (SBP, DBP) in diabetic Ren2 rats treated with either vehicle, valsartan, captopril, angiotensinogen small interfering RNA (siRNA), AGT siRNA + valsartan, or captopril + valsartan for 3 weeks. Treatment was started 15 weeks after the induction of diabetes. Days −3 to 0 correspond to the period immediately before treatment. Data are mean±SEM of n = 7–9. *P <0 .05 versus vehicle and/or indicated group. Figure S2. Effect of diabetes induction on the levels of angiotensinogen (A), renin, (B) and renin (C) in Ren2 rats after 15 weeks and the relationship between renin (D) or prorenin (E) and angiotensinogen. Data are mean±SEM of n = 47; *P < 0.05. Figure S3. Hepatic (panel A) and renal (panel B) angiotensinogen levels in diabetic Ren2 rats treated with either vehicle, angiotensinogen (AGT) small interfering RNA (siRNA) or AGT siRNA + valsartan. Treatment was started 15 weeks after the induction of diabetes. Panel C shows the immunoblots. Data are mean±SEM of n = 7–9. *P < 0.05 versus vehicle. Figure S4. Renal expression of the angiotensin II type 1a and 1b (AT1a, AT1b) receptor (A and B), Neph1 (C), nephrin (D) and podocin (E) in diabetic [file BPH-180-80-s001.pdf]
